# Isolated Nocturnal Hypertension and Target Organ Damage: A SHIP-AHOY Ancillary Study

**DOI:** 10.21203/rs.3.rs-10083118/v1

**Published:** 2026-07-03

**Authors:** Taylor Hill-Horowitz, Meredith Akerman, Michael Ferguson, Joseph Flynn, Coral Hanevold, Marc Lande, Kevin Meyers, Mark Mitsnefes, joshua samuels, Christine Sethna, Elaine Urbina, Abby Basalely

**Affiliations:** Albert Einstein College of Medicine; Boston Children's Hospital; Seattle Childrens Hospital; Rochester University; Cincinnati Children's Hospital Medical Center; Cohen Children's Medical Center; Cohen Children’s Medical Center

## Abstract

**Background:**

Isolated nocturnal hypertension (INH) and its relationship to target organ damage (TOD) is poorly studied in pediatrics. This study aims to examine the epidemiology of INH in the Study of Hypertension in Pediatrics, Adult Hypertension Onset in Youth (SHIP-AHOY) cohort and its association with TOD. INH is hypothesized to be associated with an increased risk of TOD.

**Methods:**

Data were obtained from SHIP-AHOY participants. Primary exposure was INH, defined using static thresholds (wake < 130/80, sleep ≥ 110/65) and using the 95th percentile for ambulatory blood pressure (BP). Primary outcome was left ventricular hypertrophy (LVH), defined as left ventricular mass index > 38.6 g/m^2.7^. Logistic regression evaluated the association of INH with LVH, adjusted for age, sex, cardiovascular risk factor score, and BP tolerability.

**Results:**

Using static thresholds, INH was present in 19.7% of adolescents, 61% due to systolic BP only. Age and height were greater in INH than normotension (16.1v15.4 years, P = 0.008) (168.9v166.4 cm, P = 0.036). Other demographics and anthropometric variables did not significantly differ. INH had lower estimated glomerular filtration rate (96.3v100.3 ml/min/1.73m^2^, P = 0.027). Among INH, 27% (N = 18/68) had LVH. Wake and clinic BPs were higher, but not hypertensive, in INH. INH was associated with a 2.7-fold increase in adjusted odds of LVH compared to normotension (95%CI [1.20,5.93], P = 0.016). Use of 95th percentiles resulted in a lower prevalence of INH (10%) and LVH (19%).

**Conclusions:**

INH is prevalent and associated with 2.7-fold adjusted odds of LVH compared to normotension in adolescents. Longitudinal studies are needed to characterize long-term implications of INH.

## Introduction

Isolated nocturnal hypertension (INH), a condition defined by normotensive daytime blood pressure (BP) and elevated BP during sleep, has an increasing prevalence among adolescents (1, 2). This increasing number of INH diagnoses is attributable, in part, to the 2022 American Heart Association (AHA) guidelines for pediatric ambulatory BP monitoring (ABPM). These criteria were updated to reflect data from prior Study of Hypertension in Pediatrics, Adult Hypertension Onset in Youth (SHIP-AHOY) cohort studies, which suggest that these static thresholds to delineate BP phenotypes provide an optimal sensitivity-specificity balance for the identification of target organ damage (TOD) in adolescents; whereas previous guidelines, based on 95th percentile thresholds, were too high for older and taller adolescents (3). Now with simplified BP phenotypes consistent with adult definitions, the guidelines define nocturnal hypertension in individuals aged 13 years and older as daytime BP < 130/80 mmHg accompanied by sleep BP ≥ 110/65 mmHg.

Previously, the diagnostic criteria for INH were based on 95th percentile thresholds (i.e., nocturnal BPs ≥ 95th percentile alongside normotensive daytime BPs). Prior pediatric investigations employing these percentile-based thresholds have demonstrated an association between INH and markers of TOD. This implies that elevated nocturnal BP, even without concomitant elevation in daytime measurements, may represent an early indicator of subclinical cardiovascular pathology (3–10). Adult cohorts, utilizing definitions consistent with the 2022 AHA pediatric ABPM guidelines, have consistently established a link between INH and adverse cardiovascular outcomes, including increased mortality, when compared to normotensive individuals, independent of mean 24-hour systolic BP (11–14).

The precise relationship between INH and TOD following the implementation of the 2022 AHA pediatric ABPM guidelines’ static thresholds remain insufficiently studied within diverse, well-characterized cohorts. Recent single-center investigations employing these updated definitions have not demonstrated a statistically significant association between INH and TOD in adolescents (1). These observations necessitate further investigation into the association of INH with TOD to inform therapeutic strategies and clinical guidelines (1, 2). Accordingly, this study aimed to determine the prevalence and clinical characteristics of adolescents with INH and to evaluate the association of INH with markers of TOD within the Study of Hypertension in Pediatrics, Adult Hypertension Onset in Youth (SHIP-AHOY) cohort.

## Methods

The protocol for the SHIP-AHOY study has been previously described in detail (14, 15). Briefly, SHIP-AHOY is a cross-sectional study of adolescents (11–19 years) recruited from six US clinical sites between 2015 and 2018. Adolescents with severe symptomatic stage-two hypertension, recent antihypertensive or lipid-lowering medication use, diabetes mellitus, kidney disease, or other chronic conditions were excluded.

Clinic BP measurements were obtained from the right arm following a standardized 5-minute rest period. ABPM devices were worn for 26 hours with readings performed every 20 minutes. The ABPM center in Seattle performed a quality review on each report before transmitting the deidentified data to Cincinnati Children’s Hospital Medical Center, which categorized the ABPMs and clinic BPs according to current guidelines. All study participants and their parents provided written informed consent and assent according to local institutional review board requirements. Sleep periods and ABPM tolerability (assessed on a 10-point Likert scale: 1 = most tolerable, 10 = least tolerable) were self-reported. Cardiovascular risk factor (CVRF) scores were generated by the number of risk factors for cardiovascular disease (dyslipidemia: LDL ≥ 155 mg/dL and/or triglyceride/HDL ≥ 3; obesity: BMI ≥ 95th percentile adjusted for age and sex; hypertension: systolic BP ≥ 95th percentile or > 130 mmHg in adolescents ≥ 13 years old by clinic systolic BP; insulin resistance: HOMA-IR ≥ 2.5) for each adolescent (16).

The primary exposure was INH, defined by the 2022 AHA pediatric ABPM guidelines as mean wake BPs < 130/80 mmHg with mean sleep BPs ≥ 110/65 mmHg. Isolated daytime hypertension (IDH) was defined as mean wake BPs ≥ 130/80 mmHg with mean sleep BPs < 110/65 mmHg. Sustained day-night hypertension was defined as mean wake BPs ≥ 130/80 mmHg and mean sleep BPs ≥ 110/65 mmHg (3). ABPM studies were also categorized by the 2014 guidelines, which define INH as mean wake BPs < 95th percentile with mean sleep BPs ≥ 95th percentile and hypertension as mean wake or sleep systolic or diastolic BPs ≥ 95th percentile for age and sex (10). “Dipping” referred to the 10–20% decrease in BPs expected during sleep. “Non-dipping” referred to someone whose BP decreases by < 10% during sleep (17).

The primary outcome was left ventricular hypertrophy (LVH), defined as left ventricular mass index (LVMI) > 38.6 g/m^2.7^. Left ventricular mass (LVM) was calculated from 2D-guided M-Mode images of the left ventricle at end diastole using the Devereux Eq. (18–20). LVMI is calculated as LVM/ht^2.7^ to adjust for body size without overcompensating for the adverse effect of obesity (21). Secondary outcomes include ABPM nighttime tolerability and metrics of TOD: creatinine, urinary albumin/creatinine ratio, eGFR (by CKiD U25 creatinine formula), HOMA-IR, CVRF, ratio of early diastolic transmitral flow velocity to early diastolic mitral annular tissue velocity (E/e’), arterial stiffness by pulse wave velocity, ejection fraction, global longitudinal strain, and ratio of peak to late velocities of mitral annular flow (e'/a').

### Statistical Analysis

Descriptive statistics (mean ± standard deviation or median [25th, 75th percentiles] for continuous variables; frequencies and percentages for categorical variables) were calculated comparing adolescents with normotension versus INH, INH versus sustained day-night hypertension, and normotension versus sustained day-night hypertension. Each of the pairs of two groups were compared using the chi-square test or Fisher’s exact test, as deemed appropriate, for categorical variables and the two sample t-test or Mann-Whitney test for continuous data. Univariable and multivariable logistic regression models were used to assess the association between INH and the outcome of LVH. Multivariable models were adjusted for potential confounders including age, sex, CVRF score, and ABPM tolerability. Univariable logistic regression and receiver operating characteristic analyses were used to assess the ability of daytime and sleep BP components to predict INH and LVH. A numerical measure of the accuracy of the model was obtained from the area under the curve (AUC)/c-statistic, where an area of 1.0 signifies near perfect accuracy, while an area of less than 0.5 indicates that the model is worse than just flipping a coin. The following was used as a guide for AUC: 0.9–1.0 Excellent; 0.8–0.9 Very good; 0.7–0.8 Good; 0.6–0.7 Average; 0.5–0.6 Poor. The performance of each of these measures was compared in terms of their AUC, using systolic BP night average as the reference. Since the empirical curves were constructed based on results from the same individuals, the statistical analysis on differences between curves considered the correlated nature of the data using DeLong’s test for correlated receiver operating characteristic curves.

A result was considered statistically significant at the p < 0.05 level of significance. All analyses were performed using SAS version 9.4 (SAS Institute Inc., Cary, NC).

## Results

Total enrollment in SHIP-AHOY was 478 participants; of these, 81 either withdrew voluntarily, did not meet entry criteria, or were lost to follow-up and therefore did not complete ABPM. One participant was withdrawn because of an abnormal echocardiogram showing valvular disease. ABPM data for 20 participants did not meet validity criteria. This resulted in 376 participants who contributed clinic and ABPM data for these analyses.

**Figure 1:**
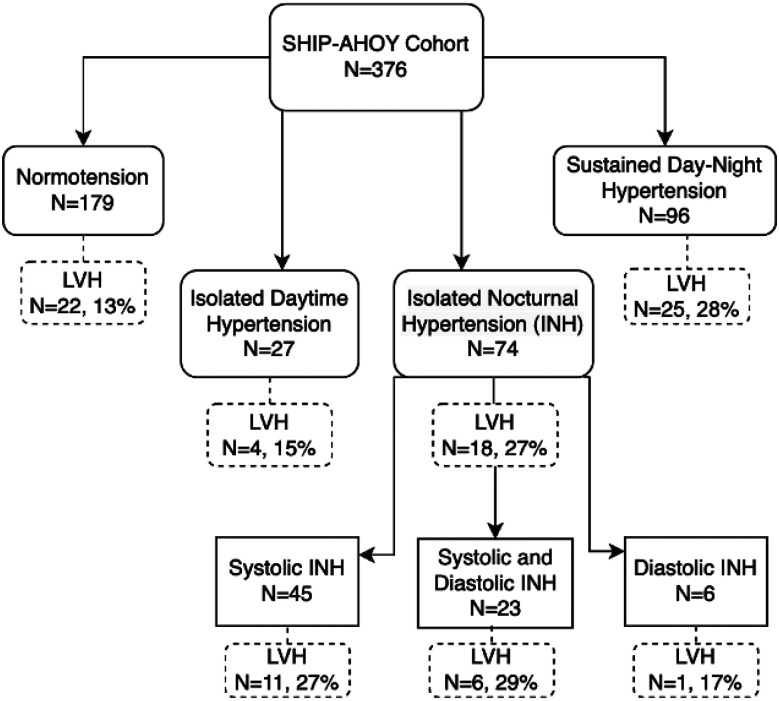
Distribution of Blood Pressure Phenotypes within the SHIP-AHOY Cohort

Within the SHIP-AHOY cohort, 74 adolescents met the criteria for INH based on the 2022 AHA guidelines, comprising 19.7% of the total cohort and 37.6% (N = 74/197) of those with hypertension. Most (60.1%, N = 45/74) adolescents with INH met the criteria for hypertension based on the systolic BP threshold alone (Fig. 1). ABPM studies tended to be less tolerable (median [IQR]: 5 [2–8] vs 3 [2–7]) (assessed on a 10-point Likert scale: 1 = most tolerant, 10 = least tolerant) in those with INH, although this difference did not reach statistical significance (Table 1). Although attenuated, diastolic dipping was preserved in the INH group (Table 1). Upon comparison of c-statistics, sleep systolic BPs were better predictors of INH than clinic or daytime systolic BPs; however, all models performed poorly (AUC ranged from 0.53 to 0.75 for all models) (Table 2).

The 2022 AHA pediatric ABPM guidelines with static thresholds identified higher prevalences of overall hypertension (52% [N = 197/376] vs 29% [N = 110/376]), INH (19.7% [N = 74/376] vs 10% [N = 36/376]), and LVH in patients with INH (27% vs. 19%) as compared to the 2014 AHA guidelines using 95th percentile thresholds (Table 3).

When comparing adolescents with INH and normotension, median age and height were greater in INH as compared to normotension (16.1 years vs 15.4 years, p = 0.022; 168.9 cm vs 166.4 cm, p = 0.039). There was no significant difference between prevalence of obesity in those with INH as compared to normotension (43.2% vs 39.1%). Clinic and mean wake systolic BPs were higher in INH, although values remained below diagnostic thresholds for hypertension (Table 1).

LVH was more prevalent in adolescents with INH (26.5%) than in normotensive adolescents (12.8%). In adjusted analyses, INH was associated with 2.7-fold increased odds of LVH (2.66, 95%CI [1.20–5.93], p = 0.016) (Table 4, Multivariate Model 3). Small but significant differences were observed in median creatinine and eGFR between INH and normotension (0.77 vs 0.68 mg/dL; 96.3 vs 100.3 mL/min/1.73m^2^) (Table 1). Other markers of TOD were not significantly different in INH vs normotension.

## Discussion

Within the multi-center SHIP-AHOY cohort, a substantial proportion (19.7%) of adolescents fulfilled the criteria for INH. Adolescents diagnosed with INH have greater age and height compared to their normotensive counterparts, with most INH diagnoses based on mean nocturnal systolic BP > 110 mmHg. Crucially, INH demonstrated an independent association with a 2.7-fold increase in the adjusted odds of LVH. To our knowledge, this investigation represents the first study to assess the associations between INH and hypertensive TOD within a cohort of otherwise-healthy adolescents.

Over one-third of participants diagnosed with hypertension presented with INH. This observation aligns with a previous single-center retrospective study that reclassified pediatric ABPM data according to the 2022 AHA guidelines, reporting INH to comprise 41% of all hypertension diagnoses (2). Conversely, another single-center investigation involving adolescents previously categorized as prehypertensive (defined by clinic BPs > 120/80 mmHg or > 90th percentile for age and sex, ABPMs < 95th percentile, and ≥ 25% load) identified INH in 55% of those reclassified to hypertension (1, 3). The absence of ABPM tolerability data in this prior study may partially account for the observed higher prevalence of INH.

The prevalence of LVH within the INH group in the present study was marginally lower (27% vs 33%) than that reported by a previous single-center review (1). This discrepancy may be attributed to the distinctive characteristics of the SHIP-AHOY cohort, which enrolled otherwise healthy adolescents presenting with normal-to-mildly-elevated clinic BP. Consistent with previous meta-analyses, which to date have predominantly utilized the 2014 AHA pediatric ABPM guidelines, INH exhibits a weaker association with LVH when compared to sustained day-night hypertension (Table 5). Specifically, the reported overall prevalence of LVH among children and adolescents with any type of hypertension is 32%, contrasted with 27% among those with INH in the current investigation. While pediatric hypertension overall conferred a 4.7-fold increased risk of LVH relative to normotension, pediatric INH was associated with a 2.7-fold increased risk of LVH compared to normotension (22). Although other markers of TOD did not significantly differ between INH and normotension, the elevated risk of LVH in the INH group, relative to normotension, suggests that the diagnostic category of INH confers a clinically meaningful increase in cardiovascular risk, although potentially less severe than the risk associated with overall hypertension. C-statistics further supported that this observed difference was primarily attributable to elevated sleep BPs, rather than the increased (though still normotensive) daytime BPs recorded in the INH cohort. This implies that the early identification of INH, prior to the manifestation of daytime hypertension, could offer an opportunity for intervention before other markers of TOD progress.

Further research is needed to characterize the long-term outcomes of INH and to ascertain whether treatment recommendations should align with those for sustained day-night hypertension. The precise nature of INH—whether it constitutes a distinct BP phenotype or represents a transitional stage for adolescents progressing from normotension to sustained day-night hypertension—remains unknown. A study by Hanevold et al. suggests that phenotype-switching is relatively common among adolescents at increased risk of hypertension, including those exhibiting elevated nocturnal BPs, reinforcing the importance of regular ABPM monitoring (23).

Given a limited sample size, the present findings should be interpreted with caution. Participants diagnosed with INH reported marginally less-tolerable nighttime ABPM, which could suggest that potential sleep disruption may contribute to elevated nocturnal BPs. Another limitation is the reliance on self-reported sleep periods; prospective studies should integrate objective measurements, such as nocturnal pulse rate, to define sleep periods. The cohort exhibited a relatively high prevalence of overweight and obese adolescents, with 40.3% classified as obese (Table 1). To account for this, obesity was adjusted for in the models through the incorporation of the CVRF score. Additionally, these metrics represent a singular cross-sectional assessment, precluding the elucidation of the longitudinal progression of adolescents’ BP phenotypes and clinical outcomes.

Despite these limitations, the present data possess several notable strengths. The SHIP-AHOY cohort is rigorously characterized and diverse, encompassing a multiethnic population drawn from six distinct clinical sites across the United States, thereby enhancing the generalizability of the findings. To facilitate a multifactorial assessment of TOD, numerous intermediate markers were quantified, including the e’/a’ ratio, global longitudinal strain, ejection fraction, and LVH. Nighttime ABPM tolerability was documented to account for potential sleep disruption induced by the ABPM procedure itself.

## Conclusions

INH affected a notable portion (20%) of the SHIP-AHOY cohort, particularly older and taller adolescents. As adolescents with INH had 2.7x odds of LVH compared to normotension, these findings suggest that the 2022 static threshold definitions may enhance the clinical utility of INH classification by identifying a greater proportion of at-risk adolescents. Adolescents with INH did not demonstrate statistically significant differences in other intermediate markers of TOD, which were elevated in adolescents with sustained day-night hypertension. Future studies should include prospective investigations evaluating whether INH precedes the development of daytime hypertension, which may inform long-term management strategies for this BP phenotype. Analysis of heart rates and activity data captured by the ABPM or other devices could be used to establish whether patients truly slept during their reported sleep periods. The impact of diastolic BPs, including the potential protective effect of preserved diastolic dipping, and elevated daytime BPs on outcomes in INH should be more thoroughly characterized in future studies.

## Figures and Tables

**Table 1:** Demographic, Anthropometric, and Clinical Characteristics of INH vs Normotension

Variable^†^	Normotension	INH	Total Cohort
	N = 179	N = 74	N = 376
Demographic and Anthropometric Characteristics			
Age*, years	15.4 (13.7–16.6)	16.1 (14.7–16.9)	15.6 (14.1–16.8)
Sex, female, N (%)	89 (49.7)	28 (37.8)	117 (46.2)
Race, N (%)			
Asian	10 (5.6)	4 (5.4)	14 (5.5)
Black	46 (25.7)	22 (29.7)	68 (26.9)
White	113 (63.1)	44 (59.5)	157 (62.1)
Other	10 (5.6)	4 (5.4)	14 (5.5)
Height,* cm	166.4 (159–174.2)	168.9 (163.2–176.6)	167 (160–174.6)
Weight, kg	71.6 (59–87.9)	77.4 (61.4–96.3)	73.3 (59.3–90.5)
BMI, kg/m^2^	25.5 (21.7–30.7)	26.7 (21.5–33.9)	26 (21.6–32)
BMI percentile	91.23 (67.7–97.9)	93.51 (58.3–98.9)	91.8 (66–98.3)
Waist/height ratio	0.49 (0.43–0.59)	0.50 (0.44–0.60)	0.49 (0.43–0.59)
Obese, N (%)	70 (39.1)	32 (43.2)	102 (40.3)
**Clinical Characteristics**			
Left ventricular hypertrophy*, N (%)	22 (12.8)	18 (26.5)	40 (16.7)
Creatinine* (mg/dL)	0.68 (0.57–0.8)	0.77 (0.69–0.88)	0.71 (0.62–0.83)
Urinary albumin/creatinine ratio	0.0035 (0.0022–0.0076)	0.0034 (0.0018–0.0056)	0.0034 (0.0021–0.0070)
eGFR* (by U25) (mL/min/1.73m2)	100.26 (90.73–112.28)	96.34 (85.84–105.86)	99.48 (89.61–110.20)
HOMA-IR	2.98 (2.00–5.70)	3.15 (2.181–5.43)	3.10 (2.07–5.65)
Cardiovascular risk factors	1 (0–2)	1 (0–2)	1 (0–2)
E/e’	5.84 (5.09–6.78)	5.82 (4.99–6.89)	5.83 (5.08–6.82)
Arterial Stiffness, m/sec	4.7 (4.33–5.3)	5 (4.5–5.43)	4.77 (4.37–5.33)
ABPM nighttime tolerability	3 (2–7)	5 (2–8)	4 (2–7)
Ejection fraction, %	58.03 (52.94–62.04)	57.29 (51.72–61.42)	57.39 (52.36–61.74)
Global longitudinal strain, %	−20.76 (−22.70–−18.69)	−20.89 (−23.42–−18.53)	−20.26 (−22.70–−18.20)
e’/a’	2.39 (1.93–2.83)	2.35 (1.96–2.91)	2.28 (1.89–2.75)
**Blood Pressure Parameters** ^††^			
Clinic SBP*	116.6 (12.6)	124.3 (9.2)	118.9 (12.2)
Clinic DBP*	69.4 (10.0)	73.3 (9.5)	70.5 (10.0)
Wake SBP*	116.0 (7.3)	122.7 (5.9)	118.1 (7.5)
Wake DBP*	68.1 (4.9)	70.19 (5.8)	68.7 (5.2)
Sleep SBP*	100.3 (6.1)	114.9 (5.8)	104.7 (9.0)
Sleep DBP*	54.0 (3.9)	61.4 (6.2)	56.3 (5.8)
SBP dipping*, %	13.4 (4.7)	6.3 (5.0)	11.4 (5.8)
DBP dipping*, %	20.5 (6.2)	12.3 (7.1)	18.0 (7.5)

* Statistically significant difference between normotension and INH

† Continuous variables represented as median (interquartile range) or mean (standard deviation) as appropriate

†† All blood pressures measured in units of mmHg

INH, Isolated Nocturnal Hypertension; BMI, Body Mass Index; eGFR, estimated Glomerular Filtration Rate; ABPM, Ambulatory Blood Pressure Monitor; SBP, Systolic Blood Pressure; DBP, Diastolic Blood Pressure

**Table 2:** C-Statistics Comparing Clinic, Wake, and Sleep Blood Pressure Prediction of Isolated Nocturnal Hypertension and Left Ventricular Hypertrophy

Model	C-Statistic [95% CI]*	
	Isolated Nocturnal Hypertension	Left Ventricular Hypertrophy**
Clinic systolic BP	0.54 [0.48, 0.62]	0.61 [0.46, 0.77]
Daytime systolic BP	0.53 [0.47, 0.59]	0.62 [0.47, 0.78]
Nighttime systolic BP	0.75 [0.71, 0.80]	0.61 [0.46. 0.76]

* Guide for accuracy of models: 0.9–1.0 “Excellent”; 0.8–0.9 “Very good”; 0.7–0.8 “Good”; 0.6–0.7 “Average”; 0.5–0.6 “Poor.”

**Table 3:** Prevalence of Left Ventricular Hypertrophy (LVH) within Isolated Nocturnal Hypertension (INH) as Defined by 2022 vs 2014 Guidelines

2014 American Heart Association Guidelines	2022 American Heart Association Guidelines
INH = 33% (N = 36/110) of hypertension	INH = 38% (N = 74/197) of hypertension
19% LVH within INH	27% LVH within INH

**Table 4:** Multivariable Logistic Regression Models of Odds of Target Organ Damage with Isolated Nocturnal Hypertension (INH) Compared to Normotension

Logistic Regression Model	Unadjusted Model		Multivariable Model 1		Multivariate Model 2		Multivariate Model 3	
	Odds Ratio [95% CI]	*P* value	Odds Ratio [95% CI]	*P* value	Odds Ratio [95% CI]	*P* value	Odds Ratio [95% CI]	*P* value
INH	2.45 [1.22–4.94]	0.012*	2.32 [1.12–4.79]	0.023*	2.49 [1.17–5.29]	0.033*	2.66 [1.20–5.93]	0.016*
Age			0.99 [0.86–1.22]	0.91	1.01 [0.80–1.25]	0.93	1.03 [0.82–1.30]	0.8
Sex			2.14 [1.02–4.49]	0.044*	2.51 [1.14–5.54]	0.022*	2.52 [1.10–5.79]	0.030*
Cardiovascular Risk Factor Score					1.38 [0.97–1.94]	0.066	1.37 [0.95–1.98]	0.092
Nighttime ABPM Tolerability							0.99 [0.87–1.13]	0.94

* Indicates P value p < 0.05 (considered statistically significant)

**Table 5:** Demographic, Anthropometric, and Clinical Characteristics of Hypertension Phenotypes

Variable^†^	Normotension	IDH	INH	Sustained Day-Night Hypertension	Total Cohort
	N = 179	N = 27	N = 74	N = 96	N = 376
**Demographic and Anthropometric Characteristics**					
Age*, years	15.4 (13.7–16.6)	15.7 (15–17.5)	16.1 (14.7–16.9)	16.0 (15.0–17.2)	15.7 (14.3–16.9)
Sex**, female, N (%)	89 (49.7)	9 (33.3)	28 (37.8)	29 (30.2)	155 (41.2)
Race, N (%)					
Asian	10 (5.6)	0 (0)	4 (5.4)	3 (3.1)	17 (4.5)
Black	46 (25.7)	8 (29.6)	22 (29.7)	26 (27.1)	102 (27.1)
White	113 (63.1)	18 (66.7)	44 (59.5)	60 (62.5)	235 (62.5)
Other	10 (5.6)	1 (3.7)	4 (5.4)	7 (7.3)	22 (5.9)
Height*, cm	166.4 (159–174.2)	170.1 (164–173.2)	168.9 (163.2–176.6)	172.4 (166–181.0)	169.0 (162.4–176)
Weight**, kg	71.6 (59–87.9)	73.5 (59.3–97.1)	77.4 (61.4–96.3)	86.6 (70.6–109.5)	75.8 (62.1–96)
BMI**, kg/m^2^	25.5 (21.7–30.7)	24.12 (21.2–32.2)	26.7 (21.5–33.9)	30.2 (24.2–35.1)	26.6 (22.4–33.0)
BMI percentile^†^	91.2 (67.7–97.9)	85.0 (59.9–98.8)	93.5 (58.3–98.9)	97.6 (84.8–98.9)	93.0 (72.3–98.6)
Obesity**, N (%)	70 (39.1)	10 (37.0)	32 (43.2)	56 (58.3)	168 (44.7)
**Clinical Characteristics**					
Left ventricular hypertrophy*, N (%)	22 (12.8)	4 (14.8)	18 (26.5)	25 (28.1)	69 (19.4)
**Demographic and Anthropometric Characteristics**					
					
Creatinine* (mg/dL)	.68 (.57–.8)	.81 (.64–.85)	.77 (.69–.88)	.73 (.61–.85)	.72 (.62–.84)
Urinary albumin/creatinine ratio	.0035 (.0022–.0076)	.0043 (.0021–.016)	.0034 (.0018–.0056)	.0035 (.0020–.007)	.0035 (.0020–.0071)
eGFR* (by U25) (mL/min/1.73m2)	100.3 (90.7–112.3)	101.9 (95.0–107.9)	96.3 (85.8–105.9)	105.5 (93.9–114.9)	100.9 (90.6–111.9)
HOMA-IR	3.0 (2.0–5.7)	3.2 (1.7–7.3)	3.2 (2.2–5.4)	4.3 (2.5–7.5)	3.3 (2.1–6.3)
Cardiovascular risk factors	1 (0–2)	1 (0–3)	1 (0–2)	2 (1–3)	1 (0–2)
E/e’	5.8 (5.1–6.8)	6.1 (4.8–7.4)	5.8 (5.0–6.9)	6.7 (5.3–7.7)	6.0 (5.1–7.1)
Arterial Stiffness, m/sec	4.7 (4.3–5.3)	5.4 (4.7–5.5)	5 (4.5–5.4)	5.4 (5.0–5.9)	5.0 (4.5–5.5)
ABPM nighttime tolerability	3 (2–7)	2 (1–7.5)	5 (2–8)	6 (2–8)	4 (2–7)
Ejection fraction, %	58.0 (52.9–62.0)	57.3 (53.3–60.7)	57.3 (51.7–61.4)	56.2 (51.3–61.4)	57.4 (52.4–61.7)
Global longitudinal strain**, %	−20.8 (−22.7–−18.7)	−20.8 (−23.2–−19.0)	−20.9 (−23.4–−18.5)	−19.0 (−22.03–−17.3)	−20.3 (−22.7–−18.2)
e’/a’ **	2.4 (1.9–2.8)	2.2 (1.8–2.6)	2.3 (2.0–2.9)	2.1 (1.7–2.5)	2.3 (1.9–2.74)

† Continuous variables represented as median (interquartile range) or mean (standard deviation) as appropriate

* Statistically significant difference between normotension and INH + normotension and sustained day-night hypertension

** Statistical significance comes from comparison between sustained day-night hypertension and normotension

IDH, Isolated Daytime Hypertension; INH, Isolated Nocturnal Hypertension; BMI, Body Mass Index; eGFR, estimated Glomerular Filtration Rate; ABPM, Ambulatory Blood Pressure Monitor

## Data Availability

The datasets analyzed during the current study can be requested from the SHIP-AHOY data coordinating center, Cincinnati Children’s Hospital Medical Center, via email to the corresponding author.

## Summary Table

**Table:**

What is known about this topic	What this study adds
Pediatric isolated nocturnal hypertension, defined by 95th percentile thresholds, has been associated with target organ damage. The relationship between isolated nocturnal hypertension defined by static ambulatory blood pressure thresholds and hypertensive target organ damage in adolescents remains uncertain.	Using static ambulatory blood pressure thresholds, isolated nocturnal hypertension was prevalent among adolescents and independently associated with increased odds of left ventricular hypertrophy. These findings support the clinical relevance of isolated nocturnal hypertension as defined by current pediatric guidelines.

## Abbreviations

INH: Isolated Nocturnal Hypertension
TOD: Target Organ Damage
SHIP-AHOY: Study of Hypertension in Pediatrics,Adult Hypertension Onset in Youth
BP: Blood Pressure
LVH: Left Ventricular Hypertrophy
AHA: American Heart Association
ABPM: Ambulatory Blood Pressure Monitoring
CVRF: Cardiovascular Risk Factor
IDH: Isolated Daytime Hypertension
AUC: Area Under the Curve
